# *Trpc2* is expressed in two olfactory subsystems, the main and the vomeronasal system of larval *Xenopus laevis*

**DOI:** 10.1242/jeb.103465

**Published:** 2014-07-01

**Authors:** Alfredo Sansone, Adnan S. Syed, Evangelia Tantalaki, Sigrun I. Korsching, Ivan Manzini

**Affiliations:** 1 Institute of Neurophysiology and Cellular Biophysics, University of Göttingen, Humboldtallee 23, 37073 Göttingen, Germany; 2 Institute of Genetics, University of Cologne, Zülpicher Strasse 47a, 50674 Cologne, Germany; 3 Center for Nanoscale Microscopy and Molecular Physiology of the Brain (CNMPB), 37073 Göttingen, Germany

**Keywords:** Amphibians, Olfactory organ, RT-PCR, *In situ* hybridization

## Abstract

Complete segregation of the main olfactory epithelium (MOE) and the vomeronasal epithelium is first observed in amphibians. In contrast, teleost fishes possess a single olfactory surface, in which genetic components of the main and vomeronasal olfactory systems are intermingled. The transient receptor potential channel TRPC2, a marker of vomeronasal neurons, is present in the single fish sensory surface, but is already restricted to the vomeronasal epithelium in a terrestrial amphibian, the red-legged salamander (*Plethodon shermani*). Here we examined the localization of TRPC2 in an aquatic amphibian and cloned the *Xenopus laevis trpc2* gene. We show that it is expressed in both the MOE and the vomeronasal epithelium. This is the first description of a broad *trpc2* expression in the MOE of a tetrapod. The expression pattern of *trpc2* in the MOE is virtually undistinguishable from that of MOE-specific *v2rs*, indicating that they are co-expressed in the same neuronal subpopulation.

## INTRODUCTION

The organization of olfactory organs varies considerably across vertebrate species. Fishes generally possess a single olfactory organ (Hamdani and Døving, 2007). Clearly anatomically segregated main and vomeronasal olfactory systems first appeared in amphibians (Taniguchi et al., 2011), and persisted in most later diverging terrestrial vertebrates including rodents (Liberles, 2014). In rodents, the main and vomeronasal systems are separated anatomically, morphologically and molecularly. Their main olfactory epithelium (MOE) contains ciliated olfactory receptor neurons (ORNs) generally expressing OR-type olfactory receptors that are endowed with the canonical cAMP-mediated transduction pathway (Liberles, 2014). Their vomeronasal organ (VNO) contains two subpopulations of microvillous receptor neurons, either expressing vomeronasal type-1 receptors (V1Rs) and Gα_i_, or vomeronasal type-2 receptors (V2Rs) and Gα_o_. Recently, an additional subpopulation of sensory neurons expressing formyl peptide receptors has been identified (for a review, see Liberles, 2014). V1R- and V2R-expressing sensory neurons depend on a phospholipase C- and diacylglycerol-mediated transduction pathway that leads to activation of canonical transient receptor potential channel 2 (TRPC2), a cation channel crucial for signal transduction in the rodent VNO. In addition, some TRPC2-independent signaling pathways are also present in the rodent VNO (for a detailed review, see Liberles, 2014; and references therein). These VNO-specific genes were first identified in rodents, but later were also found in the olfactory system of teleost fishes (for a review, see Hamdani and Døving, 2007). VR-type olfactory receptors and TRPC2 are also present in earlier diverging fishes such as sharks and lampreys (Grus and Zhang, 2009), indicating that molecular components of the rodent VNO already existed in the common ancestor of all living vertebrates. Amphibians are early diverging tetrapods compared with rodents, represent a transitional stage in the evolution of the vomeronasal system, and may thus be crucial for understanding of the evolution of the vomeronasal system and its genetic components. On the one hand, they have an anatomically segregated vomeronasal system; on the other hand, at least in the mostly aquatic *Xenopus*, expression of vomeronasal receptors is not limited to the VNO. *V1rs* (Gliem et al., 2013) and more ‘ancient’, earlier diverging, *v2rs* (Syed et al., 2013) are exclusively expressed in the MOE. Also, the cellular composition of the *Xenopus* MOE is very similar to that of the single sensory epithelium of teleost fishes (Hamdani and Døving, 2007), as it contains ciliated as well as microvillous ORNs (Gliem et al., 2013). However, the *Xenopus* VNO is already very similar to that of rodents, in the sense that it is made up solely of microvillous receptor neurons, and that its cells express *v2rs*, Gα_i_ and/or Gα_o_ (Gliem et al., 2013). In the terrestrial salamander *Plethodon shermani*, a later diverging amphibian compared with *Xenopus*, all V2Rs and TRPC2 are already confined to the VNO (Kiemnec-Tyburczy et al., 2012).

Here we identified the *trpc2* gene of *Xenopus laevis* (Daudin 1802), and found that it is expressed in cells of both the larval MOE and VNO. This is the first description of a widespread *trpc2* expression in the MOE of a vertebrate also possessing a VNO. Furthermore, we show that the expression pattern of *trpc2* in the *Xenopus* MOE is virtually undistinguishable from that of a broadly expressed *v2r* gene, *v2r-C*, suggesting a co-expression in the same subset of cells.

## RESULTS AND DISCUSSION

**Fig. 1.**
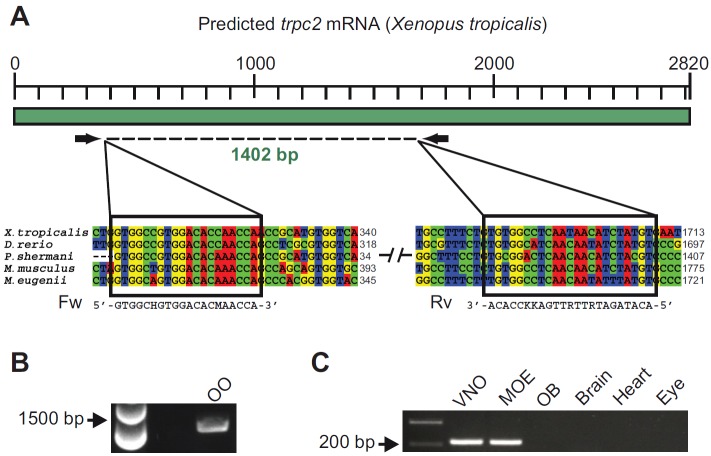
**Cloning of *trpc2* and analysis of tissue-specific expression.** (A) Schematic representation of the predicted *trpc2* transcript of *Xenopus tropicalis* and degenerate primers used for the PCR shown in B. Two fragments of the *trpc2* multi-species alignment are shown below. The black boxes highlight the conserved regions chosen to design the degenerate primers. (B) Touchdown RT-PCR with degenerate primers (see A). An amplification product of 1402 bp was detected in the olfactory organ (OO) including both the main olfactory epithelium (MOE) and the vomeronasal organ (VNO). The obtained fragment was sequenced, and in BLAST searches (http://blast.ncbi.nlm.nih.gov/) gave the best score with the predicted *X. tropicalis trpc2* sequence (90% nucleotide identity). (C) For analysis of tissue specificity, an RT-PCR (35 cycles) for *trpc2* was performed with specific primers (see Materials and methods) under stringent conditions. OB, olfactory bulb. An amplification product of the expected size was detected in the VNO and MOE, whereas no signal was detected from other organs.

*Trpc2* expression has so far not been reported in any anuran species, so we used RT-PCR to test whether the *trpc2* transcript is present in the olfactory organ of *X. laevis*. The *X. laevis* genome sequence is not available, and the *trpc2* gene sequence was not known. Therefore, we designed degenerate primers based on the *trpc2* sequence of *Xenopus tropicalis*, a species closely related to *X. laevis*. The primers were designed to target a highly conserved region among different vertebrate species (Fig. 1A). We then performed RT-PCR on the olfactory organ (MOE and VNO) of larval *X. laevis*

**List of abbreviations**

**Fig. 2.**
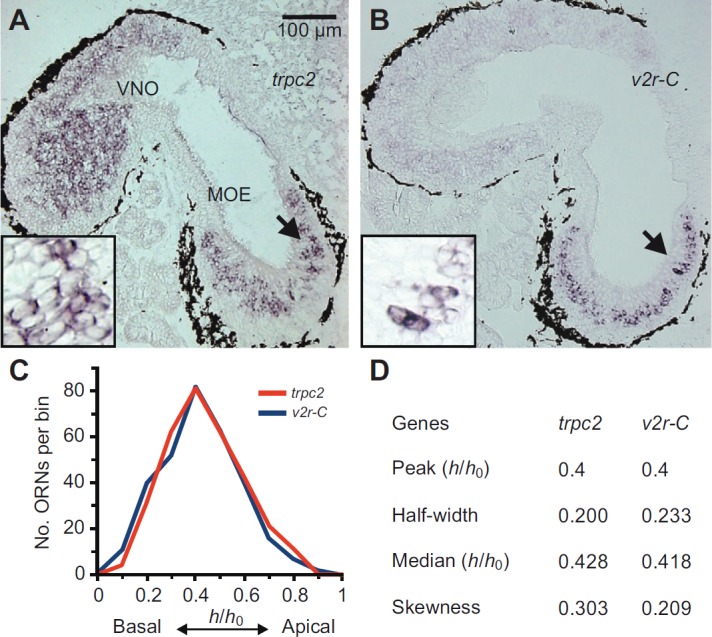
**Distribution of *trpc2*-positive cells closely mimics that of *v2r-C*-expressing cells in the MOE.** (A) Cryosections of larval *Xenopus laevis* were hybridized with antisense probes for the *trpc2* gene. The micrograph shown is from a horizontal section of larval head tissue, which contains both the MOE and the VNO. A zone of *trpc2*-positive cells was detected in the MOE and widespread labeling was visible in the VNO. The arrow is pointing at the region enlarged in the inset. (B) Cryosections of larval head tissue were hybridized with antisense probes for the *v2r-C* gene. Orientation and region as explained in A. Consistent with previous results (see Syed et al., 2013), *v2r-C*-positive cells were only found in the MOE, and occupy a discrete zone there. The arrow is pointing at the region enlarged in the inset. (C) Basal-to-apical distribution (0, most basal; 1, most apical position) of *trpc2* (314 cells, 5 sections) and *v2r-C*-expressing cells [data taken from Syed et al. (Syed et al., 2013) and shown here for comparison]. Data are given as mid-bin values (0.1 bin size); *y*-axis shows total number of cells per bin. (D) Characteristic parameters for the distribution of *trpc2*-expressing cells; values for *v2r-C* taken from our earlier work (Syed et al., 2013) are shown for comparison.

DIG: digoxigenin
MOE: main olfactory epithelium
ORN: olfactory receptor neuron
TRPC2: transient receptor potential channel 2
V1R: vomeronasal type-1 receptor
V2R: vomeronasal type-2 receptor
VNO: vomeronasal organ

and a 1402 bp fragment was isolated and sequenced (Fig. 1B). In BLAST searches, the obtained sequence (accession no. HG326501, European Nucleotide Archive) showed the *X. tropicalis* gene as the closest ortholog (90% nucleotide identity). A multi-species alignment (see supplementary material Fig. S1) showed a high degree of similarity between the *X. laevis trpc2* fragment and the sequence of diverse vertebrate species (identity: *Plethodon shermani* 78%, *Danio rerio* 72%, *Mus musculus* 72%, *Macropus eugenii* 72%). Next we analyzed the tissue specificity of the *trpc2* gene expression by performing RT-PCR with a second set of primers specific for the *X. laevis* sequence (see Materials and methods). Amplified products of the expected size were reproducibly found in the larval MOE and the VNO, whereas no signals were detected from the olfactory bulb and other organs, such as the brain, heart and eye (Fig. 1C). In a next step, the expression of *trpc2* was examined by *in situ* hybridization of larval *X. laevis* tissue sections encompassing both MOE and VNO. Numerous *trpc2*-positive cells were observed in the epithelia of both the MOE and the VNO (Fig. 2A). This bimodal expression in the two main olfactory organs is different from the situation in all other tetrapods examined so far. *Trpc2* expression in salamander (*Plethodon shermani*), the only non-mammalian tetrapod examined, is limited to the VNO (Kiemnec-Tyburczy et al., 2012), as in all mammals investigated so far (Liberles, 2014).

*Xenopus laevis* is also peculiar in that some *v2rs* are expressed in the MOE, whereas other *v2rs* are expressed in the VNO (Gliem et al., 2013; Syed et al., 2013). Thus, the TRPC2 distribution we report here parallels the distribution of V2Rs. In a terrestrial salamander, *v2r* expression is confined to the VNO, in other words, it again parallels the *trpc2* expression, which is also restricted to the VNO in this species (Kiemnec-Tyburczy et al., 2012). Such co-localization supports the hypothesis that TRPC2 is involved in V2R signal transduction.

To obtain a more stringent criterion of co-localization, we examined the relative height (in basal-to-apical direction) of cells expressing *trpc2*. This parameter shows a distinct, non-random distribution for cells expressing *v2rs* in the MOE of *Xenopus laevis* (Syed et al., 2013) (see also Fig. 2B). Evaluation of more than 300 *trpc2*-expressing cells showed that the *trpc2* gene is expressed in a distinct zone of the MOE that closely resembles the expression zone of *v2r* genes, particularly *v2r-C* (Fig. 2C,D), determined in earlier work of our group (Syed et al., 2013). In fact, the epithelial distribution of *trpc2* and *v2r-C* is almost identical, as judged by peak position, half-width, median value and skewness (Fig. 2D). Similar to *v2rs* (Syed et al., 2013), the medial-to-lateral distribution of *trpc2*-positive cells within the MOE was uniform with no tendency for lateralization (not shown). Together, these results lead to the hypothesis that in *Xenopus*, TRPC2 and V2Rs might be present in the same subpopulation of cells.

Recent work of our group showed that a large subpopulation of amino acid odor-sensitive microvillous ORNs of the *Xenopus* MOE has a phospholipase C- and diacylglycerol-mediated transduction pathway that may couple to TRPC2 (Gliem et al., 2013; Sansone et al., 2014). Microvillous ORNs in the single sensory surface of fishes are also known to be sensitive to amino acid odors and to express *v2rs* and *trpc2* (Sato et al., 2005). In fact, two fish V2Rs, OlfCa1 and OlfCc1, have been shown to be sensitive to amino acid odors (DeMaria et al., 2013; and references therein). Together, these data suggest that in *Xenopus*, TRPC2 could be involved in mediating the amino acid response of V2Rs, similar to the situation in fishes. Further investigations will be necessary to substantiate this hypothesis.

Our results strengthen the general concept that sensory neurons expressing *v2rs* and *trpc2* may be connected to the detection of non-volatile odors. In fully terrestrial vertebrates (Liberles, 2014), including a terrestrial salamander (Kiemnec-Tyburczy et al., 2012), vomeronasal receptors and *trpc2* are solely expressed in the sensory neurons of the VNO, mainly specialized for the detection of large non-volatile molecules. In contrast, in teleost fishes, vomeronasal receptors and *trpc2* are expressed in the single sensory epithelium (Sato et al., 2005; Hamdani and Døving, 2007). In the fully aquatic larvae of *X. laevis*, vomeronasal receptors (Gliem et al., 2013; Syed et al., 2013) and *trpc2* (present study) are expressed in both the MOE and VNO. It will be interesting to see whether the correlation of sensory neurons expressing vomeronasal receptors and *trpc2* for non-volatile odors holds up in adult *X. laevis*, in which the larval MOE has metamorphosed into an air nose and a new adult water nose has emerged.

Certainly the results of the present study add to growing evidence that the olfactory regionalization in *X. laevis*, and very likely also in other aquatic amphibians, is still incomplete. They possess an anatomically segregated vomeronasal system, but their main olfactory system is still very similar to that of teleost fishes, including cellular and genetic components that are already confined to the VNO in fully terrestrial vertebrates. This intermediate segregation of the *Xenopus* olfactory system results in an excellent model system to study the molecular driving forces governing the evolution of the vertebrate olfactory system.

## MATERIALS AND METHODS

### cDNA synthesis and PCR

Larvae of *X. laevis* (of either sex, stages 50 to 54) were cooled in iced water to produce complete immobility and killed by transection of the brain at its transition to the spinal cord, as approved by the Göttingen University Committee for Ethics in Animal Experimentation. Tissue samples from the VNO, MOE, olfactory bulb, brain, heart and eye were isolated and flash frozen until nucleic acid extraction. Genomic DNA and total RNA were extracted using the innuPREP DNA/RNA Mini Kit (Analytik Jena, Jena, Germany). Purity and quantity of RNA were measured using a NanoPhotometer (Implen, Munich, Germany) and integrity of RNA was evaluated using 1% agarose gel electrophoresis. cDNA synthesis was performed using the Omniscript Reverse Transcriptase Kit (Qiagen, Hilden, Germany) according to the manufacturer's protocol. Amplification of a partial sequence of the *X. laevis trpc2* gene was performed using degenerate PCR. Design of primers [5′-GTGGCHGTGGACACMAACCA-3′, 5′-ACATAGATRTTRTTGAKKCCACA-3′; modified from Kiemnec-Tyburczy et al. (Kiemnec-Tyburczy et al., 2012)] was based on multi-species alignment (ClustalW2, http://www.clustal.org/) of the *trpc2* gene sequences of *Plethodon shermani* (accession no.: JN805769), *Danio rerio* (NM_001030166), *Mus musculus* (NM_001109897), *Macropus eugenii* (GQ860951) and *Xenopus tropicalis* (predicted by automated computational analysis; XM_002941188). A touchdown PCR protocol was performed using the Phusion High-Fidelity DNA Polymerase (New England Biolabs, London, UK). The touchdown PCR parameters were: 98°C for 2 min; 20 cycles of 98°C for 1 min, 58°C for 30 s, 72°C for 1 min; 20 cycles of 98°C for 1 min, 51.4°C for 30 s, 72°C for 1 min; and 72°C for 10 min. The amplified product was then extracted from the agarose gel (QIAEXII, Qiagen), purified (QIAquick PCR Purification Kit, Qiagen) and re-amplified with the same set of primers under the same conditions. The purified product was sequenced (Seqlab, Göttingen, Germany). The sequence has been deposited in the European Nucleotide Archive (accession no. HG326501). For analysis of tissue specificity, we used the same cDNAs and a second primer pair targeting a shorter region of the *trpc2* transcript. The second primer pair was also used for producing an *in situ* hybridization probe (see below).

### *In situ* hybridization

A *trpc2* fragment of 265 bp was obtained by PCR using genomic DNA of *X. laevis* as template and 5′-AAGGGATTAAGATGGACATCAA-3′ and 5′-GCAATGCCCTTGTAGGTGTT-3′ as primers, cloned into pGEMT (Promega, Mannheim, Germany) and confirmed by sequencing. Digoxigenin (DIG) probes were synthesized according to the DIG RNA labeling kit supplier protocol (Roche Molecular Biochemicals, Mannheim, Germany) using the same forward and reverse primers with a T3 promoter site attached to their 5′ end. Tissue blocks containing VNO and MOE were fixed in 4% formaldehyde solution for 2 h at room temperature, equilibrated in 30% saccharose, and embedded in Jung tissue freezing medium (Leica, Bensheim, Germany). Sections of 8–12 μm were cut horizontally using a cryostat (CM1900, Leica). Cryostat sections were then dried at 55°C and postfixed in 4% paraformaldehyde for 10–15 min at room temperature. Hybridizations were performed overnight at 60°C using standard protocols. Anti-DIG primary antibodies coupled to alkaline phosphatase (Roche Molecular Biochemicals) and NBT-BCIP (Roche Molecular Biochemicals) were used for signal detection.

### Analysis of spatial distribution

The basal-to-apical position of *trpc2*-positive cells within the MOE was calculated by measuring the relative height of the cell, defined as distance of the center of the cell soma from the basal border of the MOE divided by total thickness of the epithelial layer at the position of the cell (*h*_rel_=*h*_cell_/*h*_layer_). The medial-to-lateral distribution of *trpc2*-positive cells within the MOE was determined by subdividing the epithelium into three parts and counting positive cells in each of the three subdivisions [for more information, see Gliem et al. (Gliem et al., 2013)]. Cell positions were measured using ImageJ (http://rsbweb.nih.gov/ij/). Median, skewness and half-width of the resulting spatial distribution were calculated from unbinned values using Open Office (http://www.openoffice.org; for more information, see Syed et al. (Syed et al., 2013)]. The epithelial position of ORNs expressing vomeronasal receptors used for comparison was determined in a previous study using identical methods (Syed et al., 2013).

## Supplementary Material

Supplementary Material
