# Supplementary material for: Trpc2 is expressed in two olfactory subsystems, the main and the vomeronasal system of larval Xenopus laevis
Source: J Exp Biol. 2014 Jul 1;217(13):2235–8. doi: 10.1242/jeb.103465 (PMC4986728; doi:10.1242/jeb.103465)
Supplement: Supplementary Material [file supp_217_13_2235__index.html]

Trpc2 is expressed in two olfactory subsystems, the main and the vomeronasal system of larval Xenopus laevis — Supplementary Material 

# *Trpc2* is expressed in two olfactory subsystems, the main and the vomeronasal system of larval *Xenopus laevis*

## JEB103465 Supplementary Material

**Files in this Data Supplement:**

- **Supplementary Material**
