# Supplementary material for: Trpc2 is expressed in two olfactory subsystems, the main and the vomeronasal system of larval Xenopus laevis
Source: J Exp Biol. 2014 Jul 1;217(13):2235–8. doi: 10.1242/jeb.103465 (PMC4986728; doi:10.1242/jeb.103465)
Supplement: Supplementary Material [file supp_217.13.2235_JEB103465.pdf]

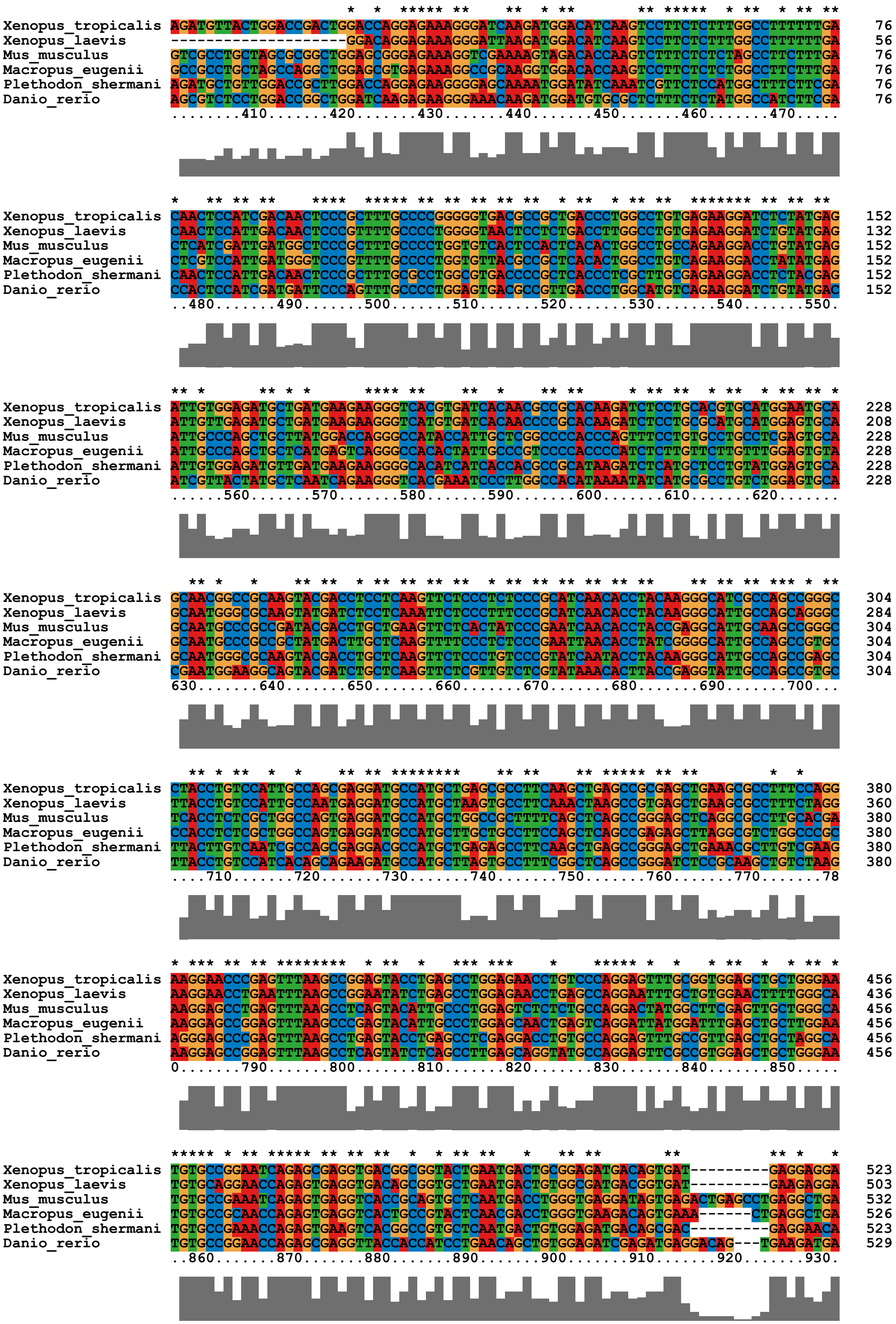

Fig. S1. Multi-species alignment of the *trpc2* gene sequence. Deduced nucleotide sequence of the partial *Xenopus laevis trpc2* gene (HG326501) aligned with the *trpc2* sequences of *Plethodon shermani* (JN805769), *Danio rerio* (NM_001030166), *Mus musculus* (NM_001109897), *Macropus eugenii* (GQ860951) and *Xenopus tropicalis* (XM_002941188). Asterisks indicate identical nucleotides in all five species. The bar diagram below the sequences indicates the degree of nucleotide conservation between species, with higher bars indicating higher conservation. The multi-species alignment was performed using ClustalW2 (http://www.clustal.org/).
